# The Effects and Mechanisms of Exercise on the Treatment of Depression

**DOI:** 10.3389/fpsyt.2021.705559

**Published:** 2021-11-05

**Authors:** Yumeng Xie, Zuotian Wu, Limin Sun, Lin Zhou, Gaohua Wang, Ling Xiao, Huiling Wang

**Affiliations:** Department of Psychiatry, Renmin Hospital of Wuhan University, Wuhan, China

**Keywords:** depression, exercise, neuroinflammation, neuroplasticity, therapeutic mechanism

## Abstract

**Background:** It is necessary to seek alternative therapies for depression, because side effects of medications lead to poor adherence and some patients do not achieve a clinical treatment effect. Recently the role of exercise as a low-cost and easy-to-use treatment for depression has gained attention with a number of studies showing that exercise is effective at reducing depressive symptoms and improving body functions such as cardiorespiratory system and cognitive function. Because of the heterogeneity of exercise therapy programs, there is no standardized and unified program. Few studies have summarized the specific properties of exercise programs (type, intensity, duration, and frequency) and clinical prescriptions for exercise are not mentioned in most articles.

**Aims:** This study aimed to investigate the feasibility and efficacy of exercise therapy for patients with depression, in order to appraise the evidence and outline accepted guidelines to direct individualized treatment plans for patients with depression based on their individual situations.

**Methods:** A systematic review of English language literature including papers published from 2010 to present in PubMed was performed. Given the feasibility of prescribing exercise therapy for patients with depression, nearly 3 years of clinical studies on the treatments of depressive symptoms with exercise were first reviewed, comparing the exercise programs utilized.

**Conclusions:** Exercise has therapeutic effects on depression in all age groups (mostly 18–65 years old), as a single therapy, an adjuvant therapy, or a combination therapy, and the benefits of exercise therapy are comparable to traditional treatments for depression. Moderate intensity exercise is enough to reduce depressive symptoms, but higher-dose exercise is better for overall functioning. Exercise therapy has become more widely used because of its benefits to the cardiovascular system, emotional state, and systemic functions.

**Recommendations:** Aerobic exercise/mind-body exercise (3–5 sessions per week with moderate intensity lasting for 4–16 weeks) is recommended. Individualized protocols in the form of group exercise with supervision are effective at increasing adherence to treatment.

## Introduction

Depression is characterized by low mood, anhedonia, and loss of interest. It has a relatively high incidence affecting both mental and physical health and places a heavy burden on individuals, families, and society. The World Health Organization once showed that ~350 million people were currently suffering from depression, and the disease may last for years (1). The Global Burden of Disease project revealed that depression was now the most serious disease burden of non-fatal neuropsychiatric diseases, and was projected to be in the top three of all disease burdens by 2030 (2). The financial toll of depression is enormous. The annual cost of days lost to depression and anxiety worldwide is estimated at $1.15 trillion, and is projected to triple by 2030 (3). The treatment and prevention of depression is a hot and widely studied research topic.

At present, depression therapies mainly include pharmacotherapy, psychotherapy, biological interventions [electric convulsive therapy (ECT), transcranial direct current stimulation, and transcranial magnetic stimulation], and naturopathic interventions. Antidepressants play a significant role in the treatment of depression, but there are disadvantages to their use, including the possibility of more and longer lasting side effects, poor adherence to antidepressant medications, increased risk of cardiovascular disease, low remission rate, and high recurrence rate (4–6). Only half of patients taking antidepressants achieve a clinical treatment effect, a decrease of 50% or more in depressive symptoms (7). In addition, dropout rates of drug therapy ranged from 15 to 132% higher than placebo (6). Concerns about antidepressant efficacy and tolerability in patients have led to interest in non-pharmacological treatments for depression (5). Compared with medication therapy, patients who are particularly averse to drugs and worry about side effects prefer non-medication therapy (5). In Steffi et al.'s interview of patients with depression (8), among the treatment options of psychotherapy, natural remedies, acupuncture, relaxation, psychotropic drugs, meditation/yoga, and ECT, psychotherapy was identified as the most favored treatment in cases of depression, with half of the interviewees identifying it as their first choice, while psychotropic drug treatment was ranked fourth. Non-pharmacological interventions may have a beneficial effect on relapse rates following treatment (9) and lower adverse effects (5). Therefore, exercise therapy, as an emerging non-pharmacological treatment, is a new feasible choice in the treatment of depression. It is simple to perform (10). Additionally, such therapy has a low cost (10, 11) and has almost no side effects (10, 11). It can prevent recurrence (12), has a positive role in overall body function (11), and has no stigma. A growing body of research has shown that exercise can relieve symptoms of depression, and that it can be used as an adjunct or individual treatment.

A systematic review of English language literature published from 2010 to present in PubMed was performed, with 822 studies searched. Terms included in the cross-searches were: depression, exercise, physical activity, swimming, aerobic exercise, non-pharmacological treatment, and exercise therapy. Randomized controlled studies, case-control studies, reviews, and animal experiments were included in our work. Inclusion criteria were: (1) treatment with an exercise program; (2) patients with depression or depressive symptoms estimated by depression scales such as the Hamilton Depression Scale, Beck Depression Inventory and the Diagnostic and Statistical Manual of Mental Disorders; and (3) the outcome measures included primary depression outcomes (cognition, mood state, and psychometric outcomes), or associated health outcomes (quality of life, anxiety, cardiorespiratory system, physiological outcomes, etc.). The literature screening process excluded articles that did not address depressive illness or depressive symptoms, and that did not include depressive symptoms and related improvement in the primary measures. Studies on prevention of depression were not included.

In the last 10 years of the literature, heterogeneity in exercise therapy programs has been found. There is no standardized and unified program. Moreover, few studies summarize the specific properties of exercise programs (type, intensity, duration, and frequency), and clinical prescriptions for exercise are not mentioned in most articles. This paper reviews observational and interventional studies incorporating both preclinical and clinical studies on the relationship between exercise and depression, including the selection and comparison of exercise programs, the effects of exercise on depression treatment and the related mechanisms, and the role of exercise in comorbid diseases with depression, its clinical application, and prospects. This paper aims to investigate the feasibility and efficacy of exercise therapy for patients with depression to appraise the evidence and outline accepted guidelines to direct individualized treatment plans for patients with depression based on their individual situations.

## The Selection and Comparison of Exercise Programs

Exercise programs are generally heterogeneous, with different types, intensities, durations, and frequencies. The types of exercise include aerobic, non-aerobic, and mind-body exercise. In clinical research, exercise types include walking (13–18), various types of bicycle use (bicycle, stationary bicycle, ergometric bicycle, and recumbent bicycle) (4, 19–30), swimming (29), jogging or treadmill use (20, 23, 24, 27, 28, 31), cross-training (22), jumping rope (22), use of “transport” machines (23), step aerobics and cardio-kickboxing (15), resistance exercises using TheraBand, dumbbells, Swiss Balls, etc. (29), stretching exercises (19, 32–34), free weight training (35), traditional Chinese exercises such as Taijiquan and Qigong (36–39), yoga (15, 32–34, 40), and Pilates (15, 41). Aerobic exercise was the most selected intervention in the experiments. To improve participants' compliance, several types of exercises were provided for participants to choose from (20). Various forms of exercise, including yoga, resistance, and aerobic exercise, have shown beneficial effects on the mental health of young people (42). Similarly, mind-body exercise, aerobic exercise, and resistance exercise are effective treatments for depressive symptoms in elderly patients (43).

In clinical trials, the most common way to determine exercise intensity is to set the amount of time each week or each day. Other studies have also utilized metabolic equivalent (MET) (44), heart rate (HR) or maximal heart rate (HRmax) (4, 14, 20, 21, 24, 44–46), maximal oxygen uptake (VO_2_ max) (4, 44, 47, 48), maximum power output (Wmax) (49), Borg Scale (16, 28), kilocalories burned per kilogram of body weight per week (KKW) (20, 24, 47), and ratings of perceived exertion (RPE) (4, 14, 24, 27, 48–50). Moderate-intensity exercise is the most popular choice in previous studies, but the standard for moderate exercise remains equivocal. In addition, high-intensity exercise programs and low-intensity exercise programs may also produce positive results. Low-intensity exercises include yoga, stretching exercises, mechanical exercises, and combined exercises, which are sometimes utilized in control groups in experiments. Moderate- and high-intensity exercise programs are often considered to have a greater antidepressant effect, but a higher intensity program often has lower compliance and a higher dropout rate (51). Hence, various factors, such as the intensity, longevity, and sustainability of exercise, need to be taken into consideration (52). There is no standardized exercise therapy program at present, and individualized treatment programs may play a role in the efficacy of exercise therapy.

In addition to a single-type exercise program, several studies (19, 53) combine different types of high-intensity and low-intensity exercises to form a systematic and structured exercise program. Similarly, one experiment (22) chose an exercise program that had both high-intensity (16–17 on the Borg Scale) and low-intensity (13–14 on the Borg Scale) exercises, resulting in improvements in the Montgomery-Asberg Depression Rating Scale (MADRS) score (mean change=-10.3, 95% confidence interval (CI) [−13.5 to −7.1], *p* = 0.038) and cardiovascular fitness (mean change = 2.4 ml oxygen/kg/min, 95% CI [1.5–3.3], *p* = 0.017) in the exercise group. The duration of the exercise program ranges from 4 to 24 weeks, with most experiments choosing 12 weeks. Another publication chose a single exercise session (17), but only showed a transient regulation effect. Only a handful of studies had follow ups at 6-month or 1-year periods to examine long-term effects, and in one 16-month work (54), patients with MDD (major depressive disorder) were followed for up to a year after 4 months of aerobic training. The results showed that regular exercise during the follow-up period seems to enhance the beneficial effects of short-term exercise and augment the efficacy of antidepressants. In a 12-month follow-up in another study (34), no increase was found in the severity of depression among patients with mild to moderate depression, suggesting that the beneficial effects of exercise on depression were not temporary. However, few studies are followed up, and more evidence is consequently needed. The frequency of exercise was based on the number of days or sessions per week, and most of the trials adapted exercise protocols with 3–5 days/sessions per week.

It is controversial whether the intensity and dose of exercise improves depressive symptoms differently. Hanssen et al. (4) demonstrated that both high-intensity low volume (HILV) (25 repetitions of 30 s high-intensity intervals at 80% VO_2_ max) and moderate continuous aerobic training (MCT) (20 min at a constant pace of 60% VO_2_ max) exercise regimens (three times a week for a period of 4 weeks) significantly reduced the depression severity index, with the former being superior. Similarly, one work has found that a higher dose (intensity, duration, and frequency) according to public health guidelines improved depressive symptoms more greatly than a lower dose of exercise (33). Compared to moderate exercise intensities, higher intensities are able to increase circulating brain-derived neurotrophic factor (BDNF) (55), a neurotrophin that is decreased significantly in depression patients. Commonly, moderate and vigorous exercise both improve the level of depression in patients with moderate depression, but very low-intensity exercise has no effect (46). A meta-analysis (56) concluded that moderate exercises, aerobic exercises, and interventions supervised by exercise professionals were more effective in MDD patients, and the benefits of exercise may have been underestimated in previous meta-analyses due to publication bias. However, other studies (33, 48) have suggested that the effect of exercise on depression is independent of the dose, intensity, or other natures of exercise. Exercise intensity may not be the main factor affecting depressive relief, as light exercise is sufficient to improve the outcome of late-life depression (25). Additionally, low-intensity mind-body exercise (such as yoga) has a significant effect as a treatment for depression (43). One probable explanation for why light exercise is more beneficial than moderate exercise is that the former is easier to perform and patients gain a greater sense of self-esteem and control (33).

This paper suggest that aerobic exercise was the most selected intervention in the experiments and moderate intensity was chosen the most though the standard for moderate exercise remains equivocal. The duration of the exercise program ranges from 4 to 24 weeks, with most experiments choosing 12 weeks. Most of the trials adapted exercise protocols with 3–5 days/sessions per week. Consequently, there is no standardized exercise therapy program at present, and individualized treatment program may play a role in the efficacy of exercise therapy. This article provides a summary table of clinical studies of exercise therapy for depression or depressive symptoms in recent years, listed in Table 1. Many of the latest studies (18, 30, 37, 38, 41, 66–69) are included.

**Table 1:** The effects of recent exercise interventions in patients with diagnosed depression.

**References**	**Sample**	**Interventions**	**Main findings**	**Possible mechanisms**	**Recommendations**
Blumenthal et al. (57)	148 MDD patients Mild to moderate severity About 51-year-olds	16 weeks aerobic exercise, 3 sessions/week, a target heart rate (HR) ranges of between 70 and 85% of HR reserve; each session lasted 45 min	•Home exercise, supervised exercise and sertraline groups decreased state anxiety scores [standardized difference = 0.3 [95% CI = −0.6, −0.04]; *p* = 0.02], compared to controls •There is no significant difference in anxiety scores in the sertraline group compared to the exercise groups (standardized difference = all exercise vs. sertraline = 0.13; 95% CI = −0.11, 0.37; *p* = 0.29)	•Exercise gives people a greater sense of engagement in life •Exercise increases feelings of control, independence and mastery, feelings of empowerment and well-being, and confidence •Physical improvements help people feel more confident and capable of positively influencing mood	•Aerobic exercise (i.e., cycling, treadmill walking or running) (≥3 sessions/week with moderate-to-vigorous intensity) is beneficial for mild to severe depression.
Schmitter et al. (58)	120 MDD adult outpatients	12 weeks running or indoor cycling, 3 sessions/week, moderate intensity (64–76% of HRmax); each session lasted 45 min	•Results may improve treatment outcomes in MDD •Results may facilitate implementation of prescriptive exercise treatment in outpatient settings		
Abdelbasset et al. (59)	69 patients with depression and heart failure Mild to moderate severity 40-60-year-olds	12 weeks aerobic exercise programs (low to moderate intensity exercise program: first 6 weeks at 40–50% of the max HR, last 6 weeks at 50–70% of the max HR; moderate-intensity exercise program: at 60–70% of max HR), 3 sessions/week; each session lasted 20–45 min	•Low to moderate intensity exercise program reduced the severity of depression (PHQ-9 scores: 16.12 ± 3.1 at baseline, 7.74 ± 3.26 at 6 weeks; 3.65 ± 1.21 at 12 weeks) •Moderate-intensity exercise program reduced the severity of depression (PHQ-9 scores: 16.34 ± 2.58 at baseline, 7.83 ± 3.22 at 6 weeks; 3.12 ± 1.18 at 12 weeks) •Non-significant differences were between the 2 exercise programs		
Imboden et al. (30)	42 patients with depression Major depression (HDRS-17 score was >16) 18-60-year-olds	6 weeks aerobic exercise, moderate intensity (60–75% of maximal heart rate), 3 sessions/week; each session lasted 45 min	•Short-term time effects were found in both groups for symptom-severity, mental toughness, physical self-description endurance score, cognitive flexibility, and body mass index (i.e., HDRS-17: AE: 22.0 ± 4.0 at baseline, 10.2 ± 6.5 at post-treatment; Control group: 20.9 ± 2.6 at baseline, 10.0 ± 7.8 at post-treatment) •Aerobic exercise (AE) was associated with comparably large depression alleviation vs. stretching exercise and with add-on benefits on working memory.		
Özkan et al. (60)	65 women with postnatal depression Pretest mean scores of the exercises (16.41 ± 1.61) and control group (15.74 ± 2.35) by Edinburgh postpartum depression scale (EPDS) 28.90 ± 4.83 years old	4 weeks exercise, ≥5 days/week, mild to severe intensity; each session lasted ≥30 min	•Exercise decreased the severity of depressive symptoms experienced in the postpartum period (Posttest EPDS: exercises group participants: 7.29 ± 1.67, control group participants: 12.54 ± 2.65) •Exercises done in the postpartum period can enable psychosocial well-being, less anxiety	•Exercise increases the level of serotonin in the brain	
Liu et al. (61)	213 centrally obese adults with depression 19–77-year-olds	24 weeks tai chi, 3 sessions/week; each session lasted 90 min	•Intervention improved physical functioning, role physical, and role emotional for the SF-36 subscales at 12 and 24 weeks [i.e., Between-group difference (95% CI): 15.1 (4.3–25.9) at 3 months; 18.0 (7.0–29.0) at 6 months (*p* < 0.01)]	•Exercise increases emotional well-being, feelings of positivity, psychological self-efficacy and functional abilities, decreases pain •Exercise reduces the secretion of corticotropin, leads to a reduction in the sympathetic activity and helps modulation of neuro-endocrine-immunologic pathways •Yoga increases the activity of the GABA system to improve mood	•Mind-body exercise (i.e., yoga, tai chi) is recommended to last longer per session than aerobic exercise. •High-dose yoga may have greater improvements on all scales than low-dose yoga.
Streeter et al. (62)	28 MDD patients Moderate severity 18-55-year-olds	12 weeks yoga, 2 sessions/week in low-dose group and 3 in high-dose group; each session lasted 90 min	•Both high-dose and low-dose group improved BDI-II scores (Low-dose group: from 27.73 ± 8.00 to 10.20 ± 10.21; high-dose group: from 24.38 ± 7.21 to 4.92 ± 4.54)		
Scott et al. (63)	32 adults with MDD 18–65-year-olds	20 weeks yoga, high-dose group (HDG) (three 90-min yoga classes and four 30-min homework sessions per week), low-dose group (LDG) (two 90-min yoga classes and three 30-min homework sessions per week)	•Significant improvements in all outcome measures (depression, anxiety, sleep quality, etc.) were found for both groups, with acute and cumulative benefits •HDG showed greater improvements on all scales (i.e., Patient Health Questionnaire: Low-dose: 4.867 ± 3.563; high-dose: 3.400 ± 2.473 at 12 weeks)		
Haussleiter et al. (64)	111 inpatients Major depression 45.05 ± 12.19 years old	6 week standardized guided exercise therapy (GET) and self-organized activity (SOA), 3 sessions/week; each session lasted 50 min	•GET was superior to SOA in reducing depression symptom severity (*p* = 0.017), specifically improving suicidality (*p* = 0.028) as well as time (*p* = 0.003) and severity of diurnal variation (*p* = 0.027)	•The effects may be associated with improvement of the immune system, control of body weight, etc. •The effects may be associated with increased serum insulin-like growth factor 1 levels	Studies of comparison classes: •The exercise program with instruction was superior to the program without instruction. •Both aerobic and stretching exercises have considerable antidepressant effects. The type of exercise may not affect antidepressant effects much.
Moraes et al. (65)	27 older patients with MDD 60–81-year-olds	12 weeks aerobic training (AT), strength training (ST), control group (CG), moderate intensity (AT: 60% of the VO_2_ max or 70% of the HRmax; ST: 70% of the maximum strength capacity; CG: low intensity), 2 sessions/week; each session lasted 30 min	•Compared with the CG, the AT and ST groups reduced depressive symptoms (i.e., HAM-D score: AT: 14.33 ± 2.82 before and 7.44 ± 2.06 after, ST: 13.44 ± 3.46 before and 8.55 ± 2.87 after, CG: 14.57 ± 1.81 before and 13.42 ± 2.07 after)		

## The Mechanisms of Exercise Therapy for Depression

It is widely known that exercise has beneficial effects on depressive symptoms and body functions. However, more research is needed into the mechanisms underlying the antidepressant effects of exercise. Most studies on the mechanisms of the antidepressant effect of exercise are rodent studies, and some clinical studies are involved as well. Therefore, we have synthesized rodent research and human studies to summarize the mechanisms of the antidepressant effect of exercise.

The mechanisms of exercise therapy for depression are related to psychological mechanisms and physiological mechanisms. The former includes psychosocial and cognitive factors; the latter includes anti-inflammatory effects, neuroplasticity mechanisms, etc. The biochemical factors involved include β-endorphin (20), vascular endothelial growth factor (VEGF) (70), brain-derived neurotrophic factor (BDNF) (20, 49, 71), 5-hydroxytryptamine (5-HT) (20, 70), insulin-like growth factor 1 (IGF-1) (70, 72), histone methyltransferase G9a (73), neuropeptide oxytocin (OXT) (73), arginine vasopressin (AVP) (73), and several others (74–77), which will be introduced in detail below.

### Psychosocial and Cognitive Factors

Person-centered physical therapy approaches (person-centered aerobic exercise using intervals with higher perceived intensity for 10 weeks) (22), in which investigators explored the patient's perceived needs and beliefs based on humanism, typically have a better outcome in treating depression. This finding, as well as the significant effects of mind-body exercise on depression mentioned above, suggested the role of psychological factors in the improvement of depressive symptoms through exercise, at least to some extent.

Combining the results of previous studies, exercise can enhance self-worth and self-esteem after receiving positive feedback (78), and may augment self-efficacy, self-confidence, sleep quality, and life satisfaction (20, 26, 79, 80). Physical therapist-guided aerobic exercise enhanced the feeling of being alive and made them feel that they were doing something good for themselves (79). A review (81) that conducted non-systematic searches to identify mechanisms of the antidepressant effect of exercise provided evidence that exercise can promote self-esteem, self-perception, and self-efficacy and can enhance social support to create a buffer against depressive symptoms. For cognitive function, a 12-week, multimodal exercise program of 70–90% HRmax may play a role in transferring negative thoughts of youth with MDD (53). Some studies (80, 82) also suggested that during exercise interventions, patients may seek social support, which reduces loneliness. Based on the psychological and social benefits, group exercise is generally considered to be more effective than individual exercise, but no studies have compared their efficacies to each other (individual compared to group exercise). In addition, exercise may also be related to mechanisms involved in behavioral activation (53).

### Anti-inflammatory and Anti-oxidant Factors

Neuroinflammation is well-known to play a key role in the pathogenesis of depression. In the central nervous system (CNS), microglia are best known as the key immune cells of the brain (83). Findings have shown that activation of microglia under pathological conditions leads to a decrease in neurogenesis (84) and the release of a large number of inflammatory factors, including interleukin-18 (IL-18), interleukin-1β (IL-1β), interleukin-6 (IL-6), and tumor necrosis factor-α (TNF-α), which exerted neurotoxic effects and cause depression and anxiety (85). A large number of studies have confirmed that stress can cause the activation of microglia and the release of proinflammatory factors (85, 86). Caspase-1 is the effector protein of the nucleotide-binding and oligomerization domain (NOD)-like receptor (NLR) protein 3 (NLRP3) inflammasome, a member of the NLR family of cytosolic pattern recognition receptors (87). The inflammasome is known to consist of the NLR family member NLRP3, the apoptosis-associated speck-like protein containing a caspase recruitment domain (ASC) adaptor protein and the effector protein caspase-1. It has been revealed that after exposure to stressors, assembly of the NLRP3 inflammasome increases, leading to an increase in the protein levels of ASC and cleaved caspase 1 (88). NLRP3 continuously cleaves IL-1β and IL-18 precursors into mature IL-1β and IL-18 through activated caspase-1, thereby producing a large number of downstream inflammatory mediators (89). After caspase-1 is activated, cleaved caspase-1 is released from the inflammasome (90). Thus, we proposed that exercise alleviates depressive symptoms through antidepressant effects, although the mechanism of exercise therapy for depression has not been fully elucidated.

The anti-inflammatory effect of exercise has been mentioned in articles (12, 53, 72, 74, 75). Sixteen-week exercise (dose recommendations based on the Ainsworth Compendium of Physical Activities) with at least four 40-min homework exercise sessions per week, consisting of at least moderate physical activity, increases the production of the anti-inflammatory cytokine IL-10 and reduces the production of proinflammatory cytokines, proinflammatory markers, and cytokines such as C-reactive protein (CRP) and IL-6 in MDD, which may be the mechanism of its antidepressant effect of depression (75). A clinical investigation by Ventura et al. (76) showed that aerobic exercise programs, of which in-clinic training consisting of a combination of moderate intensity aerobic exercises (1-min intervals) and moderate to high intensity strength and calisthenic conditioning (1-min intervals) for 45 min twice a week over a period of 6 months, can reduce IL-6 levels in patients with schizophrenia, and that the reduction of pro-inflammatory IL-6 level was significantly associated with reduced depressive symptoms, suggesting that exercise plays an important role in the anti-inflammatory mechanism in the treatment of depression. More research is needed to illustrate the anti-inflammatory effect of exercise.

Oxidative stress (OS)-related endothelial damage is associated with the occurrence and development of a variety of diseases, such as vascular depression and late-life depression (91). Moderate aerobic exercise reduces OS and inflammation, reduces endothelial damage, improves endogenous antioxidant defense systems, and promotes the expression of superoxide dismutase, glutathione peroxidase, and glutathione reductase. It also reduces the concentration of several inflammatory biomarkers, such as IL-6, homocysteine, and tumor necrosis factor-α (TNF-α), and restrains the activity of NADH oxidase (74, 91). Brocardo et al. (92) illustrated that voluntary wheel running alleviated depression-like symptoms in male rats from prenatal ethanol exposure and that the positive effects of exercise were linked to increased levels of antioxidants such as glutathione. Moreover, an animal study (93) has demonstrated that exercise increased the endothelial nitric oxide synthase (eNOS) expression and activation both in the vascular wall and in the perivascular adipose tissue, which was associated with lowering ROS in the vascular wall, leading to the improvement of the vascular function. In addition, the inhibition of hippocampal DNA oxidation (11) was also mentioned. In clinical studies, 12-week aquatic exercise in depressed elderly individuals (*n* = 92) was shown to reduce depression and anxiety, and decreases oxidative stress (94).

### Neurogenesis and Neuroplasticity

The antidepressant effects of exercise are associated with enhanced neurogenesis in the adult hippocampus, increased synaptic growth, and synaptic plasticity (47, 74, 95, 96), which effectively stimulate neurogenesis in the hippocampal dentate gyrus (DG) and promote hippocampal growth (12, 97). Within the signaling pathways, two related growth factors are VEGF and IGF-1 (70). Research by Tang et al. (98) indicated that exercise increases the expression of oligodendrocyte-related proteins and the number of oligodendrocytes in the hippocampus of rats with depression. In previous studies, growth-associated protein 43 (GAP-43) and synaptophysin (SYN) were involved in the alterations of hippocampal plasticity in mice, and SIRT1/microRNA, CREB/BDNF, and AKT/GSK-3β signaling pathways may be related to the regulation (77). Exercise also promotes neural differentiation, enhances the plasticity of new neurons by increasing the complexity of dendrites and the short-term synaptic plasticity of some nerves, and has pro-neurogenic action of progenitor cells of the hippocampus (95). Ma et al. (96) examined the roles of physical exercise in inducing hippocampal neurogenesis in humans and rodents to determine its effectiveness in the prevention or decline of cognition, confirming that physical exercise induces neurogenesis including proliferation, differentiation, survival, maturation, and function.

BDNF is associated with neuronal survival and synaptic plasticity (17), and a growing body of research suggests that BDNF may mediate exercise to improve depressive-like behavior. Szuhany and Otto's studies of outpatients with MDD and persistent depression (99) demonstrated that both acute and regular exercise caused an increase in BDNF. Several additional studies (49, 71) have also linked BDNF to neurogenesis and plasticity. A study of elderly women with major depression showed that a single exercise session significantly increased serum BDNF levels; however, it showed a significant secondary decrease in BDNF serum levels after 30 min of rest (17), suggesting that acute exercise might be beneficial for MD treatment and further studies including the effects of long-term exercise on BDNF serum levels are needed. However, to date there are no reports of sustained augmentation of BDNF after acute exercise (74). Differences between studies suggest that more longitudinal studies are needed to demonstrate the relationship between exercise and BDNF.

In addition, exercise causes an increase in neurotransmitters associated with increased activity of dopamine, 5-hydroxytryptamine, and noradrenaline in the central nervous system (CNS) (28, 74, 100). The volume and structural integrity of the hippocampus, anterior cingulate cortex (ACC), prefrontal cortex, and corpus callosum and alteration of the responsivity of the ventral striatum are also related to the neural mechanism of exercise therapy for depression (100, 101). A more recent study (102) also confirmed that moderate-intensity (which was defined as 50–70% of maximum heart rate) aerobic exercise, consisting of 30 min running, 4 days per week for 3 months, can induce structural changes in the rostral ACC in adolescents with subthreshold mood syndromes.

### Other Physiological Factors

The relationship between exercise and the hypothalamic pituitary adrenal (HPA) axis is complex and is influenced by the different natures of exercise and stress and the characteristics of the research population (74). There is experimental evidence that short-term running stimulates neurogenesis in rats, while long-term exercise may be turned into a stressor by the activation of the HPA axis, which reduces the rate of cell proliferation (97).

Swimming can alleviate the depression-like behavior of rat offspring, which is associated with mitochondrial movement related to O-GlcNAc transferase (OGT), which includes the PINK1/Parkin and AKT/GSK3 signaling pathways. Another study (103) has also shown that this effect was related to the improvement of mitochondrial morphology and function, which illustrated that hippocampal ATP production and mitochondrial membrane potential of rats in the exercise group increased significantly, while the production of mitochondrial superoxide decreased significantly; also, the high HPA activity in rats with depression was reversed. Endurance exercise has also been shown to stimulate mitochondrial biogenesis in a wide range of tissues (74).

The neuroimmune mechanisms of exercise include an increase in the number of macrophages entering the CNS in cellular immunity, the upregulation of CXCL1, CXCL12, MKP-1, and IGF-1 in humoral immunity, and the activation of anti-inflammation and antioxidation, as detailed in Harris Eyre's review (104). The downstream changes were as follows: (1) HPA axis: glucocorticoids (GCs) were downregulated, and glucocorticoid receptor (GR) receptor activity and density increased. (2) Neurodegenerative changes: neuronal proliferation increased, metabolism decreased, BDNF was upregulated, and caspase activation decreased. (3) Monoamine metabolism: 5-HT and NA were upregulated, and indoleamine-2,3-dioxygenase (IDO) and quinolinic acid (Quin) were downregulated.

The antidepressant effects are related to hormonal responses. The mechanisms involved include increased testosterone levels (11) and endorphins release (46) as well. Resistance exercise has been shown to elicit a significant acute hormonal response, involving hormones including testosterone, growth hormones (GH), cortisol, insulin-like growth factors (IGFs), insulin, catecholamine, and other hormones, as detailed in Kraemer's review (105).

It is also believed that exercise regulates the metabolism of kynurenine, which enhances antidepressant ability (106–108).

## The Therapeutic Effect of Exercise on Depression

The beneficial effects of exercise on depression and depressive symptoms have been demonstrated in the last decade, with a large body of research supporting the effect of physical exercise on reducing depressive symptoms in patients (26, 95). For instance, a study (30) aimed at patients with depression (aged 18–60) revealed regardless of aerobic exercise or stretching exercise, there was a significant short-term time effect for symptom severity [Hamilton Depression Rating Scale 17 (HDRS17): *p* < 0.001, η^2^ = 0.70; BDI: *p* < 0.001, η^2^ = 0.51], mental toughness (*p* < 0.001, η^2^ = 0.32), physical self-description endurance score (*p* = 0.013, η^2^ = 0.16), cognitive flexibility (*p* = 0.013, η^2^ = 0.14), and body mass index (BMI) (*p* = 0.006, η^2^ = 0.19). A meta-analytical review (109) that included 1,452 clinically depressed adults revealed that large effects in favor of exercise were found, irrespective of the exercise mode and study quality, compared to the control condition.

A summary of clinical studies over the past 3 years on the exercise effects on depression is provided in Table 1. Aerobic exercises (i.e., stride walking, treadmills, cycling, cross trainers) for people with other conditions (schizophrenia, dementia, chronic stroke, at high risk of depression, etc.) (18, 66, 72, 107, 110–124) and mind-body exercises (i.e., yoga, tai chi, Qigong, Ba-Duan-Jin, Pilates) for people with other conditions (healthy state, menopause, aging, scleroderma, Parkinson's disease, fibromyalgia, HIV, etc.) (37, 39–41, 120, 125–136) were proven to improve depression, anxiety, cognitive function, and overall functions such as sleep quality, psychological well-being, sexual function, and cardiorespiratory fitness as well. Although aerobic exercise and mind-body exercise are the most studied types of exercise with significant results, resistance exercise, stretching exercise, endurance exercise, and other types of exercise have also proven to be effective treatment options for depression (67, 72, 107, 110, 137–140). Structured/combined exercise (including resistance, aerobic, strength, balance, relaxation, and endurance exercise) is recommended (53, 69, 141–151); however, there have been no studies comparing structured exercise with a single exercise program.

Existing studies have almost covered patients with depression in a wide range of characteristics. Most of the research subjects were adults (18–65 years old), and a few subjects were teenagers and elderly people. Exercise benefits young people in terms of improving their mental health, and there is a bidirectional relationship between physical activity and adolescent mental health (42). Nasstasia et al. (53) used a structured multimodal exercise program combined with motivational interviewing (MI) as adjunctive therapy in patients aged 15–25 with major depression (MDD), resulting in a significant difference in the revised Beck Depression Inventory II (BDI-II) cognitive and affective subscales of the intervention group after treatment and improved somatic health and behavioral activation. Several studies of patients with late-life (older than 65) depression (152) have also confirmed that exercise reduced depressive symptoms.

There are a number of trials (29, 153–155) that have failed to prove the efficacy of exercise in treating depressive symptoms. For instance, clinical research in 2012 has shown that the provision of tailored advice and encouragement for physical activity did not improve depression outcomes or antidepressant use in adults with depression when compared with usual care (156). In Table 1, there are two experiments that failed to reveal the antidepressant effect of exercise. One study (157) reported that a sedentary program may be more beneficial to boys' moods than exercise. Another study (158) reported that 1-week high cadence cycling failed to improve depression symptoms. Methodological weaknesses, excessively severe depressive symptoms in participants, discrepancies in the mean age of the sample, imperfect exercise programs, and bias would affect the results.

### The Role of Exercise in the Treatment of Depression—As a Single Therapy, an Adjuvant Therapy, or a Combination Therapy

Exercise can be used as a single treatment for depression (47). As a single therapy, one clinical trial (24) reported that depressive symptoms were significantly reduced in MDD patients (58%) after 8 weeks of moderate aerobic exercise, compared with 22% in the placebo group.

As a therapy to treat depression, exercise is an intervention with moderate and significant effects that can be used as an independent approach and as an adjuvant to antidepressant therapy (159). Aerobic exercise has been performed as an independent intervention and as an adjunct intervention to medication and psychotherapy (160). For patients with major depression who are resistant to medication, moderate intensity exercise as an adjunct intervention can ameliorate depressive and functional parameters (13). For MDD patients, the addition of exercise therapy to sertraline therapy was associated with a higher rate of depression relief and a significant improvement in symptoms than the sertraline-only group, with improvements observed from the end of the 4th week of exercise (161, 162). As adjunct treatments to pharmacotherapy for major depressive disorder (MDD) in older persons, the aerobic training and strength training groups showed significant reductions in depressive symptoms (treatment response = 50% decrease in the pre to post intervention assessment) through the Hamilton Depression Rating Scale and Beck Depression Inventory, compared with the control group (65). In a clinical publication (4) aiming to investigate the effects of different exercise modalities on the depression severity index and arterial stiffness, the results confirmed the positive effects of aerobic exercise as adjuvant treatment for patients with depression. In addition, the combination of exercise and sertraline treatment has a positive effect on cardiac autonomic control, reducing cardiovascular risk (163). A recent review (95) suggests that fluoxetine and running may have similar but not completely overlapping ranges of antidepressant effects and may therefore effectively complement each other to relieve depression- and anxiety-like symptoms. These findings further support the effectiveness of exercise as an adjunct to antidepressant therapy.

Cognitive behavior therapy (CBT) is one of the three major schools in the field of psychotherapy. The combination of exercise and CBT has a greater improvement for mild to moderate depression, suicidal ideation, and activities of daily living than CBT alone (16), and an exploratory randomized controlled trial reported that the combination of CBT and exercise had an anti-inflammatory effect by increasing IL-10 and reducing CRP in patients with major depression (75). The initial phase of cognitive activation allows patients to find the benefits of exercise on a psychological level and thus increase their motivation to exercise, which in turn benefits patients with depression more, making this is a benign combination. In addition, there is preliminary evidence (160) that suggests the feasibility of combining repetitive transcranial magnetic stimulation (rTMS) with aerobic exercise.

Evidence suggests that exercise programs can improve depressive symptoms and overall functioning as an adjunct to traditional therapies such as medication or psychotherapy.

### Comparison of Exercise Therapy With Other Therapies or Between Exercise Therapies

Exercise therapy is believed to be equivalent to psychotherapy and medication therapy (32). A meta-analysis (159) showed that when compared with established psychotherapy (cognitive behavioral therapy, interpersonal psychotherapy, and cognitive therapy) or antidepressant treatment, there was no difference in the therapeutic effect of exercise. Another meta-analysis (164) specifically compared exercise therapy and selective serotonin reuptake inhibitor (SSRI) antidepressant treatment and found no difference in efficacy. Chronotherapy is the application of temporal pharmacology to improve efficacy and reduce adverse reactions. Exercise therapy is less effective than chronotherapy in patients with major depression (165), but both therapies are viable treatments, with depressive symptoms continuing to be improved and remitted during long-term follow-up. In addition to the comparisons between exercise therapy and traditional treatment methods such as medication and psychotherapy, there are some studies comparing exercise methods with different doses or different programs (4, 63–65, 166–173). The following information can be obtained from existing studies: The benefits of exercise therapy are comparable to traditional treatments for depression (168–170). In general, moderate intensity exercise is enough to reduce depressive symptoms, but higher doses are better for overall functioning (4, 63, 166). Mind-body exercise or aerobic exercise under supervision are recommended, and they are superior to stretching exercise and breathing exercise (30, 64, 171, 172).

### The Role of Exercise in Depression Combined With Other Diseases

Currently, an increasing number of people focus on combined depression because the prognosis of the patient is generally worse when disease is combined with depression. As a treatment for these patients, exercise has a considerable beneficial effect (174). For example, exercise reduced the depressive symptoms of cancer patients with primary lung cancer, chemotherapy cancer, and gastroesophageal junction cancer (14, 19, 107). While reducing the depressive symptoms of diabetes and other chronic diseases, exercise can also reduce cardiovascular risk factors and improve psychological status and the quality of life (36, 175). For clinical heart failure patients, exercise has obvious benefits for depressive symptoms, and the beneficial effects are consistent regardless of age, intervention duration, exercise settings, and test quality (78). In addition, exercise has a certain degree of curative effect on depressive symptoms and the overall function of patients with schizophrenia, Alzheimer's disease, Parkinson's disease, hemodialysis, arthritis, and other rheumatisms, heart transplantation, multiple sclerosis, etc. (10, 95, 140, 176–179). In prenatal and postpartum depression, exercise is an effective treatment method, which has been confirmed by several studies (82, 180). Consequently, exercise therapy has become more widely used because of its benefits to the cardiovascular system, emotional state and systemic functions. The advantages of exercise therapy are reflected when the body has contraindications to drugs.

### Additional Effects of Exercise

Exercise as an optional treatment for depression can not only improve depression symptoms and reduce their severity, but also has a positive effect on the overall function of the body, cardiorespiratory fitness (45, 181), and cognitive function (24, 26). As mentioned earlier, the combination of structural physical exercise and sertraline treatment may be beneficial to the autonomous cardiac control of elderly MDD patients (163). Another large sample study (44) showed that the combination of exercise and sertraline can also improve the risk factors for coronary heart disease in patients with MDD and reduce the risk of atherosclerotic cardiovascular disease (ASCVD). In addition, exercise improves arterial stiffness (4).

Many depressed patients have persistent symptoms after taking antidepressants, such as cognitive impairment. One investigation (182) explored the effects of different doses of exercise on the cognitive function of MDD patients and concluded that the cognitive function of participants in the high-dose group [16 kcal per kg per week (KKW)] was significantly improved compared to that of the low-dose group (4 KKW). In both groups, depressive symptoms and cognitive functions in multiple areas, such as psychomotor speed, visuospatial memory, and executive function, were improved. On the other hand, a systematic meta-analysis by Sun et al. (183) reported that exercise failed to show significant effects on the overall cognitive function and individual cognitive domains of MDD patients. However, interventions that combine physical exercise with cognitive activities can significantly enhance overall cognitive function. Low-intensity exercises show a more positive effect than high-intensity exercises, which may be related to better compliance with low-intensity exercises. There is direct evidence that exercise can improve the performance of cognitive tasks such as spatial memory, pattern separation, situational fear adjustment, and new object recognition (95). The multiple beneficial effects of exercise therapy make its clinical application more valuable.

## Future Development and Application of Exercise to the Treatment of Depression

One of the first ideas that suggested exercise as a preventative and therapeutic approach to depression was proposed by Brown (184), but there were no clinical studies of exercise therapy in depression at that time. Since the 1990s, there has been a growing awareness of the link between exercise and mental health. There is also a growing body of research on the effects of exercise on physical and mental illnesses such as depression and anxiety. In 1992, an investigation confirmed that high-intensity aerobic exercise had positive effects on well-being in an adolescent population (185).

Currently, the relevance of the exercise to prevent or treat depression is well-established. However, limitations still need to be solved. Due to the heterogeneity of exercise programs and methods for evaluating curative effects selected in different studies, more high-quality studies are needed to explore standard exercise programs, the comparison of therapies, and the efficacy of combined use. Longer follow-ups are also required to determine the long-term efficacy of exercise. The mechanism of exercise treatment for depression also needs further research and exploration. A relatively large number of patients are not willing to participate in the exercise treatment because they lack motivation, and the feasibility of regular exercise training at home under less supervision has not been clarified at this time (4). The use of behavioral activation (186), mobile app support (4), and a short duration of physical activity (187) are promising tools to increase motivation and adherence to regular exercise. Group and supervised exercise patterns were more likely to increase motivation (113). Severe depression may be better treated with antidepressants as an initial therapy (5).

The World Health Organization (WHO) and the UK National Institute for Health and Clinical Excellence (NICE) guidelines recommend physical exercise as the standard complementary treatment option for depression (109). Among previous depression treatment guidelines, the suggestion proposed by NICE in 2009 recommended physical exercise as the initial treatment for mild to moderate depression (154). In Rethorst's review of the antidepressive effects of exercise, it was suggested that exercise be conducted three times a week for 45–59 min per session, lasting for 10–16 weeks (188).

Currently, clinical exercise prescriptions have not yet been widely used. The reasons why doctors do not generally prescribe exercise include lack of infrastructure (58%), belief that exercise prescriptions are not effective (50%), that patients do not follow the prescription due to habit (50%), and that patients are in poor physical condition (33%) [see questionnaires of Zanetidou et al. (25)]. After participating in the study, most doctors changed their views, with 80% of them prescribing medications for elderly patients to treat their depressive symptoms. In addition to the aforementioned questionnaire results, Murri et al. (189) also concluded the following common views: exercise has a positive effect on the body (“from the neck down”), but its effectiveness in treating the core features of depression (“from the neck up”) is not fully understood. It is believed that only strenuous exercise is effective and that exercise is harmful to the elderly. Therefore, some stereotypes hinder the large-scale application of exercise prescriptions. Currently, the beneficial effects of exercise are increasingly recognized, and more exercise is prescribed.

## Prospects and Conclusions

At present, people pay more attention to lifestyle, as a healthy lifestyle improves many disease symptoms. Exercise is expected to be popular in the clinical treatment of depression in the future, and the following points should be considered when prescribing exercises in clinical practice: on the one hand, the individual exercise program should be customized based on the patient's age, gender, exercise ability, financial ability, and value synergistically. On the other hand, the patient's personal preference should be foremost. Additionally, the exercise plan should be supervised or instructed by professionals such as physical therapists, personal trainers, and other health professionals providing regular guidance or special support.

Exercise has therapeutic effects on depression in all age groups (mostly 18–65 years old), as a single therapy, an adjuvant therapy, or combination therapy, and the benefits of exercise therapy are comparable to traditional treatments for depression. Moderate intensity exercise is enough to reduce depressive symptoms, but higher-dose exercise is better for overall functioning. Exercise therapy has become more widely used because of its benefits to the cardiovascular system, emotional state and systemic functions. Aerobic exercise/mind-body exercise (3–5 sessions per week with moderate intensity lasting for 4–16 weeks) is recommended. Individualized protocols in the form of group exercise with supervision are effective at increasing adherence to treatment.

## Author Contributions

YX, ZW, and GW participated in the design of the study, carried out the data collection, and drafted the manuscript. LS and LZ helped with the analysis. LX and HW advised on the article ideas and helped to draft the manuscript. YX and GW edited the manuscript. All authors read, edited, and approved the final manuscript.

## Funding

This study was supported by the National Natural Science Foundation of China (Nos. 81871072 and 82071523) and the Medical Science Advancement Program of Wuhan University (No. TFLC2018001). Design of this study was supported by the Key research and development program of Hubei Province (2020BCA064).

## Conflict of Interest

The authors declare that the research was conducted in the absence of any commercial or financial relationships that could be construed as a potential conflict of interest.

## Publisher's Note

All claims expressed in this article are solely those of the authors and do not necessarily represent those of their affiliated organizations, or those of the publisher, the editors and the reviewers. Any product that may be evaluated in this article, or claim that may be made by its manufacturer, is not guaranteed or endorsed by the publisher.
